# Hybrid collagen–cellulose–Fe_3_O_4_@TiO_2_ magnetic bio-sponges derived from animal skin waste and Kenaf fibers for wastewater remediation

**DOI:** 10.1038/s41598-023-40520-y

**Published:** 2023-08-17

**Authors:** E. F. Assanvo, S. Nagaraj, D. Boa, P. Thanikaivelan

**Affiliations:** 1 Advanced Materials Laboratory, CSIR-Central Leather Research Institute (CSIR-CLRI), Sardar Patel Road, Adyar, Chennai, 600 020 India; 2 Laboratoire de Thermodynamique et de Physico-Chimie du Milieu, UFR SFA, Université Nangui Abrogoua, 02 BP 801, Abidjan 02, Côte d’Ivoire; 3 University of Madras, Chepauk, Chennai, 600005 India

**Keywords:** Nanobiotechnology, Environmental chemistry, Photocatalysis, Green chemistry, Composites

## Abstract

Water pollution from synthetic dyes and oil spills has a significant impact on the environment and living species. Here, we developed a low-cost, environmentally friendly and easily biodegradable magnetic hybrid bio-sponge nanocomposite from renewable resources such as collagen and cellulose (Kenaf fibre cellulose–collagen, KFCC). We loaded it with magnetic bimetallic Fe_3_O_4_@TiO_2_ (BFT) NPs to produce a photocatalyst material (KFCC-BFT) for the treatment of colored wastewater as well as a sorbent for oil–water separation. The characterization of the bimetallic BFT NPs by XRD, HRTEM and VSM showed the deposition of TiO_2_ particles onto the surface of Fe_3_O_4_ with lattice interlayers spacing of 0.24 and 0.33 nm for Fe_3_O_4_ and TiO_2_, respectively with ferromagnetic property. The UV–vis diffuse reflectance spectra result indicated that the band gap energy of bio-sponges decreases with the increase of the bimetallic moiety. The photocatalytic efficiency of the as-prepared magnetic hybrid bio-sponge in the degradation of crystal violet dye was up to 91.2% under visible light conditions and 86.6% under direct sunlight exposure. Furthermore, the magnetic hybrid bio-sponge was used to separate motor oil from water (> 99%) and had a high oil sorption capacity of 46.1 g/g. Investigation of the recyclability and reusability performance for 9 cycles revealed that the bio-sponge had a high sorption capacity for up to 5 cycles. Our results suggest that the bio-polymer-supported BFT hybrid nanocomposite is a cost-effective and easily biodegradable photocatalyst and has great potential for real-field environmental remediation applications.

## Introduction

Environmental pollution, predominantly water pollution, is currently a major global threat imposing severe hazards to both human and ecosystem health. Water pollution is a severe concern for all stakeholders including society, public authorities, and industries^1^. The rapid development of industries such as textile, leather, paint, paper, printing, and plastic as well as offshore oil and gas leads to the increase in the discharge of various pollutants into water^2^. One of the most common pollutants are organic pollutants such as organic dyes, agrochemicals, phenols, cosmetics, pharmaceutical and petrochemical wastes, which are very hazardous to aquatic organisms and damaging whole ecosystems due to the reduction of the amount of dissolved oxygen in the water in their oxidative decomposition process^3,4^. Amongst various organic pollutants, soluble dyes and insoluble oils are causing severe damage to the ecosystem. Synthetic dyes are the most used in industries and are composed of polyaromatic molecules containing one or more azo bonds N=N–) that give permanent color to materials. The application of synthetic dyes is diverse ranging from textile and paint to leather industries^5–7^. The unused synthetic dyes present in water are toxic, carcinogenic, and mutagenic and may affect the aquatic environment and human beings, even at a low-level concentration^5,6^. It is challenging to completely remove colour pollutants from water using traditional wastewater treatment techniques such as absorption and oxidation^8,9^.

In general, effluents containing soluble contaminants, solids, colloids, organic matter and minerals are removed through various physical, chemical and/or biological techniques^10^. For the removal of organic contaminants from contaminated effluents, conventional water treatment techniques such as air flotation, precipitation, coagulation, oxidation, adsorption, ion exchange, membrane filtration, etc., are popular^11,12^. Each treatment method has its own advantages and limitations such as operational costs, efficiency, functionality, dependability, eco impression, post-treatment requirements, and also the creation of sludge and toxic by-products. For intense, photocatalytic degradation has been considered an effective and advanced approach to removing the dyes from the wastewater owing to its advantages such as low cost, non-selective degradation and operating with conventional separation technology without further secondary pollution^13^. Among different semiconductor photocatalysts developed for the removal of organic contaminants in wastewater, TiO_2_ nanoparticles (NPs) have been extensively used owing to non-toxicity, low cost and availability, excellent chemical and thermal stability, high catalytic activity, and excellent carrier properties^14–16^. However, the TiO_2_ NPs have some drawbacks that restrict their real field uses as photocatalysts for wastewater treatment, including agglomeration, the high recombination rate of photo-generated electron–hole pairs and tough recovering due to smaller particle sizes^17–19^. Moreover, the intrinsic large bandgap energy (3.23 eV) limits the use of TiO_2_ NPs for solar irradiation applications^20^. Bimetallic Fe_3_O_4_@TiO_2_ (BFT) NPs have been extensively investigated to overcome the separation or recovery problem of TiO_2_^21^. Magnetic Fe_3_O_4_ NPs based photocatalysts provide a real field practicable method to recover magnetic particles from the reaction media as well as possible reuse of the catalyst. Additionally, magnetic Fe_3_O_4_ NPs have superior magnetism, excellent compatibility, low cytotoxicity despite high loading and can speed up the transfer of photo-induced electrons between (Fe^3+^, Fe^2+^) to increase the photocatalytic activity of TiO_2_^22,23^.

Water pollution by oil spills can happen during the process of offshore activity by accidents involving tankers, barges, pipelines, refineries, drilling operations, and storage facilities. Oil spills in water bodies not only destroyed aquatic organisms such as sea birds, mammals, algae, etc. but the sand on the coastline was also severely polluted. Following a large oil spill, the effects on ecosystems and economies can last for decades, making the problem of oil/water separation a global challenge^24–27^. Several methods can be used to control and eradicate oil leaks from water such as oil bloom or containment bloom, which is a temporary floating barrier method used to contain oil, a skimmer using skimming agents to skim oil from the surface of the water, in situ burning to fire away oil slicks at the surface of the water, chemical dispersants to break the emulsion oil/water and physical sorbents to absorb oil. Among the aforementioned methods for oil/water separation, physical sorbents have gained special attention amongst researchers and industry communities owing to their low density, high 3D porous material (sponges or aerogels), large surface area, surface roughness, tortuosity, recyclability, complete removal and absence of secondary pollution^28–31^. Hence, an effective photocatalytic method for the removal of both dyes and oils from wastewater is ideally required.

In recent years, native cellulose and naturally derived nano cellulose have been extremely used in research as renewable and sustainable materials for the preparation of sorbent sponges suitable for the photodegradation of organic pollutants and oil/water emulsion separation. Due to its numerous beneficial features such as renewability, biodegradability, lightweight, large surface area, good mechanical strength, thermal stability and non-toxicity, extracted cellulose from the lignocellulosic fiber is frequently used as filler or reinforcing material for the preparation of composites, paper and textile industries, automobiles and 3D scaffold biomaterials. Super-hydrophobic and super-hydrophilic/super-oleophobic cellulose sponges have been fabricated for oil/water separation^26,28,29^. In the same trend of preparing bio-based advanced materials, collagen which is a by-product waste from the leather industry has been recently used for the preparation of sorbent sponges for wastewater treatment. Collagen-based biomaterials have been prepared for various applications including scaffolds for tissue engineering^32–34^ bioengineered scaffolds for scarless healing of deep burns^35^, conducting hybrid aerogels^36^, and water remediation^6^ due to its wide availability, high biocompatibility and biodegradability. Each year, a huge amount of collagen-containing waste is disposed into the environment from leather industries, therefore, preparing environmentally free and cost-effective bio-based materials from leather waste is highly feasible and viable.

Herein, a hybrid nanocomposite comprising cellulose and collagen biopolymeric matrices embedded with bimetallic Fe_3_O_4_@TiO_2_ NPs was prepared with multifunctional properties for environmental remediation applications. Magnetic bimetallic Fe_3_O_4_@TiO_2_ NPs were synthesized by mixing the pre-synthesized Fe_3_O_4_ NPs with titanium precursor followed by chemical reduction. Cellulose was extracted from Kenaf fibers (Kenaf fiber cellulose, KFC) whereas collagen was extracted from cowhide trimming wastes and combined to form a Kenaf fiber cellulose–collagen (KFCC) matrix. Combining BFT with KFCC yielded bio-based Kenaf fiber cellulose–collagen nanocomposite sponge incorporated with BFT NPs (KFCC-BFT) was characterized for their structural and functional properties and further studied for wastewater remediation applications.

## Materials and methods

### Materials

Kenaf fiber was extracted from the kenaf tree (cultivated traditionally by farmers for making ropes to tie piles of rice after harvest for storage) collected in Issakaha village, from the Korhogo region, Côte d’Ivoire (Ivory Coast). All the plant experiments were in compliance with relevant institutional, national and international guidelines and legislation. Freshly flayed hides was purchased from the local slaughterhouse, Chennai, India. Raw cowhide trimming wastes were collected from the leather processing unit at CSIR-Central Leather Research Institute, Chennai, India. Hide powder was prepared from raw cowhide trimming wastes and were treated using the conventional process to remove the unwanted hair and flesh based on our previous report^37^. All chemicals were of analytical grade and were used without any further purification. Chemicals used were sodium hydroxide (SD Fine Chem Limited, 98%), sodium chlorite (LOBA Chemie, 80%), sodium sulfite anhydrous (EMPARTA, 98%), acetic acid (SRL, 99.9%), acetone (SD Fine Chem Limited), citric acid, titanium (IV) isopropoxide (Sigma Aldrich, 97%), iron (II) sulphate (SD Fine Chem Limited, 99%), iron (III) sulphate (SD Fine Chem Limited, 75%), ammonia (Rankem), isopropanol (Qaligens, 70%), ethanol (Hayman, 100%) and nitric acid (SD Fine Chem Limited, 69–72%).

### Extraction of cellulose from Kenaf fibers

Kenaf fibers were dried and cut into small pieces. 20 g of dried fibers were boiled with 300 mL of 6% NaOH (w/v) for 1 h. The pre-treated fibers were washed and ground in a laboratory mixer for 3 min to produce pulp-like fibers. Then, the delignification process was carried out by bleaching the pulp-like fibers with 300 mL 0.7% (w/v) sodium chlorite at pH 4, adjusted with 5% acetic acid and boiled for 3 h. The partially lignin-free fiber residue was washed with distilled water to neutral pH. The neutralized fiber residue was boiled with 300 mL 5% (w/v) sodium sulfite for 5 h to remove the lignin completely as well as hemicellulose partially. The obtained holocellulose was boiled with 300 mL 17.5% (w/v) sodium hydroxide solution for 5 h to remove the hemicelluloses. The isolated cellulose was washed with distilled water until neutral pH and air-dried^38^.

### Synthesis of magnetic BFT nanoparticles

#### Synthesis of Fe_3_O_4_ nanoparticles

The Fe_3_O_4_ NPs were synthesized by the co-precipitation method. Briefly, 250 mL of the Erlenmeyer flask containing 100 mL of 0.1 M FeCl_3_ and 100 mL of 0.05 M FeSO_4_.7H_2_O were stirred for 30 min at 60 °C. Then, 25% of ammonia solution was added drop by drop until the pH reaches 10, leading to dark yellow precipitate. The obtained Fe_3_O_4_ NPs were washed thoroughly with distilled water to neutral pH and separated using centrifugation for 20 min at 10,000 rpm. The purified NPs were dried in a hot air oven at 100 °C for 8 h and calcinated at 500 °C for 2 h under a nitrogen environment.

#### Synthesis of TiO_2_ nanoparticles

For the synthesis of TiO_2_ NPs using the sol − gel method, 5 mL of titanium (IV) isopropoxide was added to 20 mL of ethanol and the mixture was stirred at 500 rpm at 60 °C for 30 min. Then, a mixture of 20 mL of ethanol/water (1:1) and 2 mL of HNO_3_ (72%) was added dropwise. The resulting mixture was stirred at 1000 rpm for another 3 h at 100 °C to form a gel. The obtained product was centrifuged at 10,000 rpm for 20 min. The collected pellet was washed with distilled water and then with ethanol and kept at 100 °C in a hot air oven for 8 h and calcinated at 500 °C for 2 h under a nitrogen environment.

#### Synthesis of magnetic BFT nanoparticles

Magnetic bimetallic Fe_3_O_4_@TiO_2_ NPs were synthesized through the chemical reduction method. In this procedure, 5 mL of titanium (IV) isopropoxide was dissolved in 25 mL isopropanol. Subsequently, 0.2 g of the pre-synthesized Fe_3_O_4_ NPs were added under vigorous stirring at 600 rpm for 1 h at 80 °C. To this, 100 mL of 0.1 M citric acid solution was added dropwise under continuous stirring at 300 rpm for 3 h to yield brown color precipitate (magnetic bimetallic Fe_3_O_4_@TiO_2_ NPs). The obtained magnetic NPs were purified by centrifugation at 10,000 rpm for 20 min, washed with ethanol and kept at 100 °C in a hot air oven for 8 h and calcinated at 500 °C for 2 h under a nitrogen environment.

#### Fabrication of hybrid magnetic Kenaf fiber cellulose–collagen sponge embedded with bimetallic Fe_3_O_4_@TiO_2_ NPs (KFCC-BFT)

The weighed amount of organic fibers (collagen and kenaf fiber cellulose) in 1:1 proportion was dissolved in 90 mL of 0.5 M glacial acetic acid using a homogenizer (Ika, T25) at 4 °C for 10 min. The wt.% of magnetic BFT NPs was calculated based on the weight of the organic fibers and three bio-sponges were fabricated namely KFCC, KFCC-BFT5, and KFCC-BFT10 corresponding to 0, 5 and 10% (wt./wt.) of BFT in the mixture. The BFT was sonicated using Lark Probe Sonicator for 2 min and added to the organic collagen–cellulose mixture. The mixture was homogenized at 4 °C for 1 h and transferred into 12 well plates and kept in a freezer (− 40 °C) for 2 h. Then, frozen products were kept in a lyophilizer (Delvac freeze drier) for 24 h to obtain hybrid magnetic KFCC-BFT bio-sponges.

#### Crystal violet degradation

The photocatalytic activity of the hybrid bio-sponges was investigated by analyzing the degradation of water-soluble crystal violet (CV) dye under visible light and sunlight irradiation under ambient environmental conditions. The initial concentration of CV is 2.5 × 10^−5^ mol/L. The photodegradation reaction was carried out in a 100 mL glass beaker containing 30 mL of CV dye. Before the photodegradation reaction, the glass beaker containing 30 mL of CV dye and bio-sponge was stirred for 30 min in the dark to establish adsorption–desorption equilibrium. Then, the solution was irradiated using 200 W Hg (Xe) with a medium pressure lamp (66,454 Newport Oriel Instrument) at room temperature or exposed directly to sunlight at ambient temperature for 180 min at neutral pH. The glass beaker holding the sample was held 20 cm from the light source during the irradiation procedures. At regular intervals of 10 min, samples of up to 1 mL were taken out for UV–visible examination.

#### Motor oil removal

The oil adsorbency of the nanocomposite was determined according to ASTM: F726-12 method^39^. A weighed dry bio-sponge (m_0_) was placed in a beaker containing excess used motor oil and allowed to immerse for 15 min. Then, the sponge was removed and drained for 2 min and the wet weight was taken (m_1_). The oil adsorbency as the ratio of oil adsorbed to the dry sponge weight was calculated as below.

1 $$Oil\,adsorbency=\frac{m}{{m}_{0}}$$

With m_0_ the initial dry sponge weight, m_1_ weight of the sponge at end of the oil test and m = (m_1 _− m_0_) net oil adsorbed. The recyclability and reusability performance of the hybrid bio-sponge was also investigated according to ASTM:F726-12 using the centrifugation method. The centrifugation was carried out at 5000 rpm for 5 min at 25 °C. Additionally, the bio-sponge was added to a petri dish containing excess distilled water and 0.5 g of used motor oil in order to test the sponge's ability to absorb oil from an oil–water mixture and track the sponge in a magnetic field. With the help of a strong magnet, the sponge was moved through the petri-dish for 15 min to adsorb the oil. Then, the sponge was removed and drained for 2 min and the adsorbed oil was squeezed out. Digital photographs were taken during each stage of the process.

### Characterization

The crystalline structure of TiO_2_, Fe_3_O_4_ and BFT NPs were examined by using a Rigaku Miniflex (II) desktop diffractometer with Ni filtered Cu Kα radiation source (λ = 1.5418 nm) in the angular range of 20° to 80° at goniometer speed of 5 °C /min at room temperature. To identify the phase of the samples, XRD results were uploaded to the Joint Committee on Powder Diffraction Standards (JCPDS) software and the report was generated. The average crystallite d-size of NPs was determined using Debye–Scherrer Equation.

2 $$D=\frac{k\lambda }{\beta cos\theta }$$

where, k represents the constant (0.89), λ stands for the wavelength of X-ray (1.5418 nm), β denotes full width at half the maximum of the diffraction peak and *θ* is the Bragg angle. Dynamic light scattering measurements (DLS) of the prepared NPs were analyzed using Zetasizer Nano ZS, series ZEN 3600, Malvern Instruments to obtain the particle size. UV–vis spectra of the NPs were obtained using a UV–visible spectrophotometer (Shimadzu UV-1800). The spectrum was taken from 200 to 800 nm with a 0.5 nm scanning interval. UV–vis diffusion reflectance spectra (UV–vis DRS) of bimetallic NPs and bio-sponges were obtained in a UV–Vis–NIR spectrophotometer (Agilent technologies) to determine the band gap energy.

The morphologies of the samples were examined with a field emission scanning electron microscope (FESEM, TESCAN, model-CLARA, Czech Republic) equipped with an energy-dispersive X-ray (EDX) spectroscopy. For high-resolution transmission electron microscopy (HRTEM, JEOL JEM 3010) analysis of BFT NPs, 5 µL of the sample was dispersed in 1 mL HPLC grade ethanol using water bath sonication for 30 min and 20 µL of the dispersed NPs was coated over the copper grid and dried at room temperature. The sample was observed at 200 kV voltage using LaB6 filament. To analyze the surface compositions of the nanocomposites, X-ray photoelectron spectroscopy (XPS) was performed using the ESCALAB 250 xi, Thermo Scientific. While pore size and pore diameter were determined using the Barrett–Joyner–Halenda (BJH) approach, the surface area was estimated using the Brunauer–Emmett–Teller (BET) theory. Fourier-transformed infrared (FT-IR) spectroscopic analysis was carried out using a Jasco spectrometer (Model No-4700) for the extracted cellulose, collagen, and synthesized bio-sponge samples. The surface wettability of sponges was characterized by water contact angle and oil (hexadecane) contact angle with a 5µL droplet. The measurements were obtained with a contact angle meter (Holmar, series HO-IAD-CAM-01) at room temperature. The magnetic properties of BFT, KFCC-BFT5 and KFCC-BFT10 were measured at room temperature using a vibrating sample magnetometer (VSM, Lakshore VSM 7410S).

## Results and discussion

### Characterization of TiO_2_, Fe_3_O_4_ and magnetic BFT NPs

**Figure 1:**
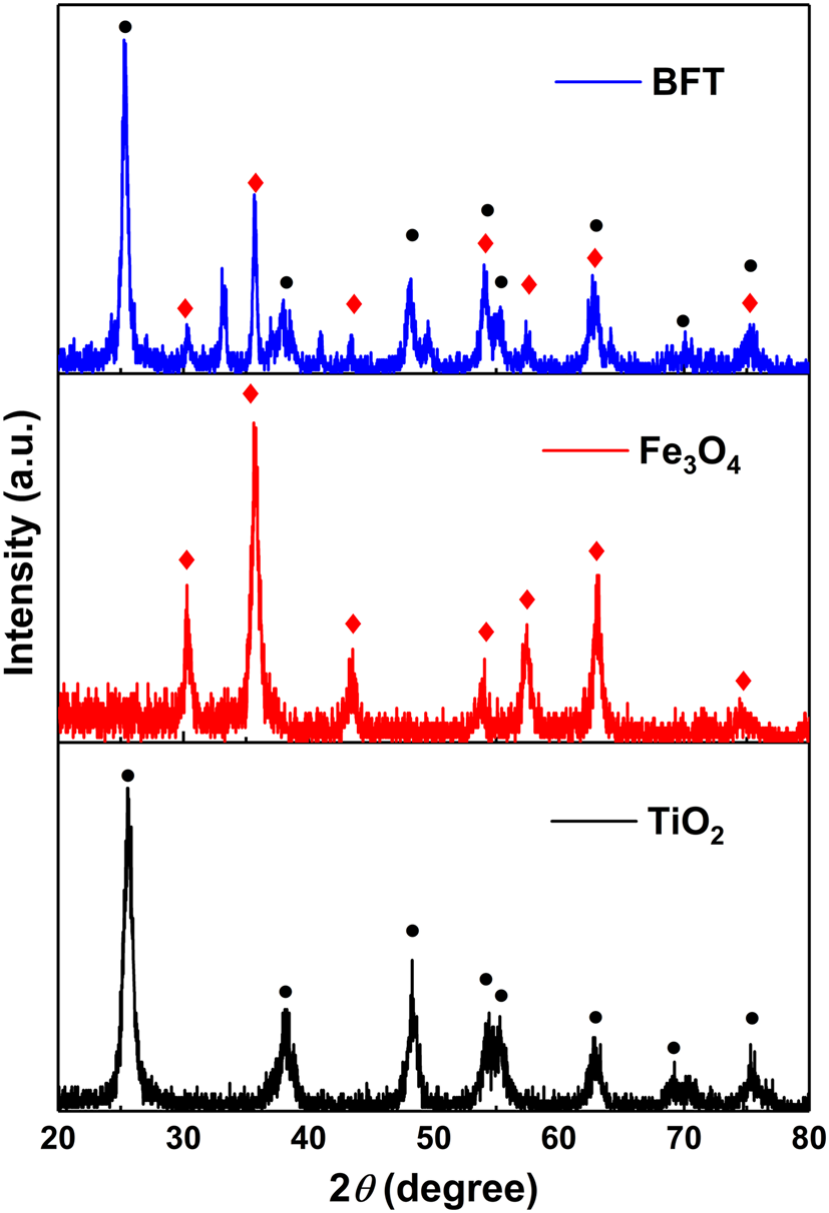
X-ray diffractograms of the synthesized nanoparticles namely (**a**) TiO_2_, (**b**) Fe_3_O_4_ and (**c**) magnetic BFT.

Figure 1 shows the XRD patterns of the synthesized NPs. The diffraction peaks at 2θ angles of 25.4°, 37.8°, 48.1°, 54.1°, 55.3°, 62.9°, 68,7°, 70.5° and 75.3° can be assigned to the (101), (004), (200), (105), (211), (204), (116), (220) and (215) crystal planes of the anatase TiO_2_ phase (JCPDS No.021-1272)^40^. The magnetite NPs exhibit diffraction peaks at 2θ angles of 30.2°, 35.6°, 43.3°, 53.7°, 57.5°, 62.9° and 74.5° corresponding to the (220), (311), (400), (422), (511), (440) and (533) crystal planes of cubic Fe_3_O_4_ phase (JCPDS No. 65-3107)^41^. The sharp and intense crystallinity peaks at 2θ angles of 25.4° and 35.6° corresponding to the (101) and (311) planes of TiO_2_ and Fe_3_O_4_, respectively are present in the diffraction pattern of magnetic BFT NPs along with all the other peaks of both the nanoparticles. Crystallite size of the Fe_3_O_4_ and TiO_2_ was calculated as 24 and 15 nm, respectively using Scherrer’s formula as given in Eq. 2. Furthermore, the interplaner spacing value was calculated as 0.25 and 0.34 nm, which was resulting from the intense peak of the Fe_3_O_4_ (311) plane and TiO_2_ (101) plane, respectively. However, it is interesting to note the intensity of the peaks corresponding to the Fe_3_O_4_ is lower than those of TiO_2_. This could be due to the higher amount of TiO_2_ present in the bimetallic NPs compared to the Fe_3_O_4_.

**Figure 2:**
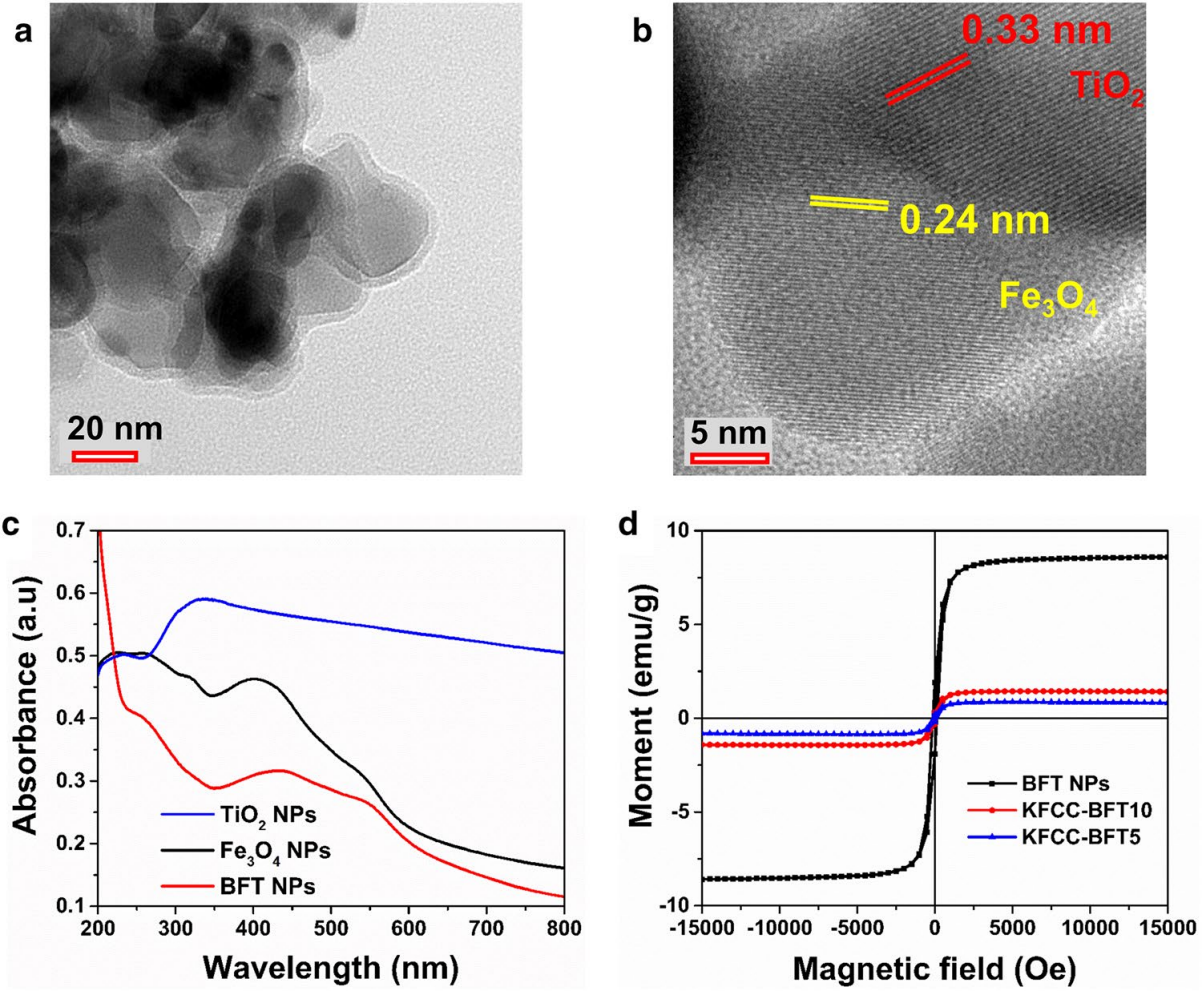
(**a**) Low and (**b**) high magnification HRTEM images of the magnetic BFT NPs. Lattice interlayer spacings originated by the (101) crystal planes of TiO_2_ and (311) crystal planes of Fe_3_O_4_ are clearly observable in the high magnification HRTEM image. (**c**) UV–vis spectra of pristine and BFT NPs and (**d**) M-H curves of BFT10 (powder Fe_3_O_4_@TiO_2_) and hybrid bio-sponges (KFCC-BFT5, KFCC-BFT10) analyzed using VSM.

The morphology of the magnetic BFT NPs was investigated by HRTEM. Figure 2a shows randomly dispersed Fe_3_O_4_ NPs coated with the TiO_2_ NPs. The result suggests that the magnetic BFT NPs are in aggregated form in contact with one another rather than the typical core–shell configurations. The Fe_3_O_4_ and TiO_2_ particles present in the bimetallic NPs seem to in the order of 20–25 nm although they are in contact aggregates. The particle size range observed here is comparable to the pristine Fe_3_O_4_ and TiO_2_ NP crystallite size obtained from XRD data. The HRTEM image (Fig. 2b) indicates that the lattice interlayer spacing of Fe_3_O_4_ and TiO_2_ is 0.24 and 0.33 nm, respectively coinciding with the (311) and (101) planes of the respective oxides, as revealed by XRD patterns^42^. The average particle size of TiO_2_ NPs, Fe_3_O_4_ NPs and uncalcined BFT NPs measured through DLS is 41, 250 and 391 nm, respectively as shown in Figure S1. After calcination at 500 °C for 2 h, it seems that BFT NPs got agglomerated with the increase in particle size to 703 nm. Overall, the particle size observed by DLS is much larger than the values obtained by XRD and HRTEM owing to the interaction of water molecules on the NP surface during DLS measurement. The UV–Vis absorption spectra of pristine TiO_2_, Fe_3_O_4_ and BFT NPs are shown in Fig. 2c. The absorption band of pure TiO_2_ NPs is in the range of 300–350 nm, which is in the UV region, while the magnetite Fe_3_O_4_ NPs exhibit the absorption band at 350–450 nm between UV and visible regions. In contrast, it should be noted that the BFT NPs are characterized by a wide band between 350 and 530 nm, which appears relatively high absorption in the visible region (400–800 nm). The addition of magnetite moiety induces a red shift in the absorption edges to a longer wavelength compared to pristine TiO_2_. Fe_3_O_4_ promotes the movement of electrons from the valence band to the conduction band, thus leading to a shift in TiO_2_ absorption in the range of its photocatalytic response^5,17^.

Bimetallic NPs and magnetic hybrid organic–inorganic bio-sponges were characterized for their magnetic behavior using room temperature magnetization by VSM. Figure 2d shows typical S-like curves with perfect saturation magnetization indicating the ferromagnetic behavior of the bimetallic NPs and the bio-sponges. Compared to pure BFT NPs (with a saturation magnetization of 8.60 emu/g), the saturation magnetization values of bio-sponges were reduced to 1.44 and 0.86 emu/g, respectively for KFCC-BFT10 and KFCC-BFT5 nanocomposites as a function of dosage of BFT. The hysteresis loops are shown in Figures S2a and b. It is seen that both the pure BFT NPs and bio-sponges exhibit coercivity of about 118 Oe. Whereas the remanent magnetization for the BFT NPs is about 1.4 emu/g and 0.16 and 0.24 emu/g for the KFCC-BFT5 and KFCC-BFT10 nanocomposites, respectively, which behaves similar to the saturation magnetization trend.

### Characterization of KFCC, KFCC-BFT5, and KFCC-BFT10

**Figure 3:**
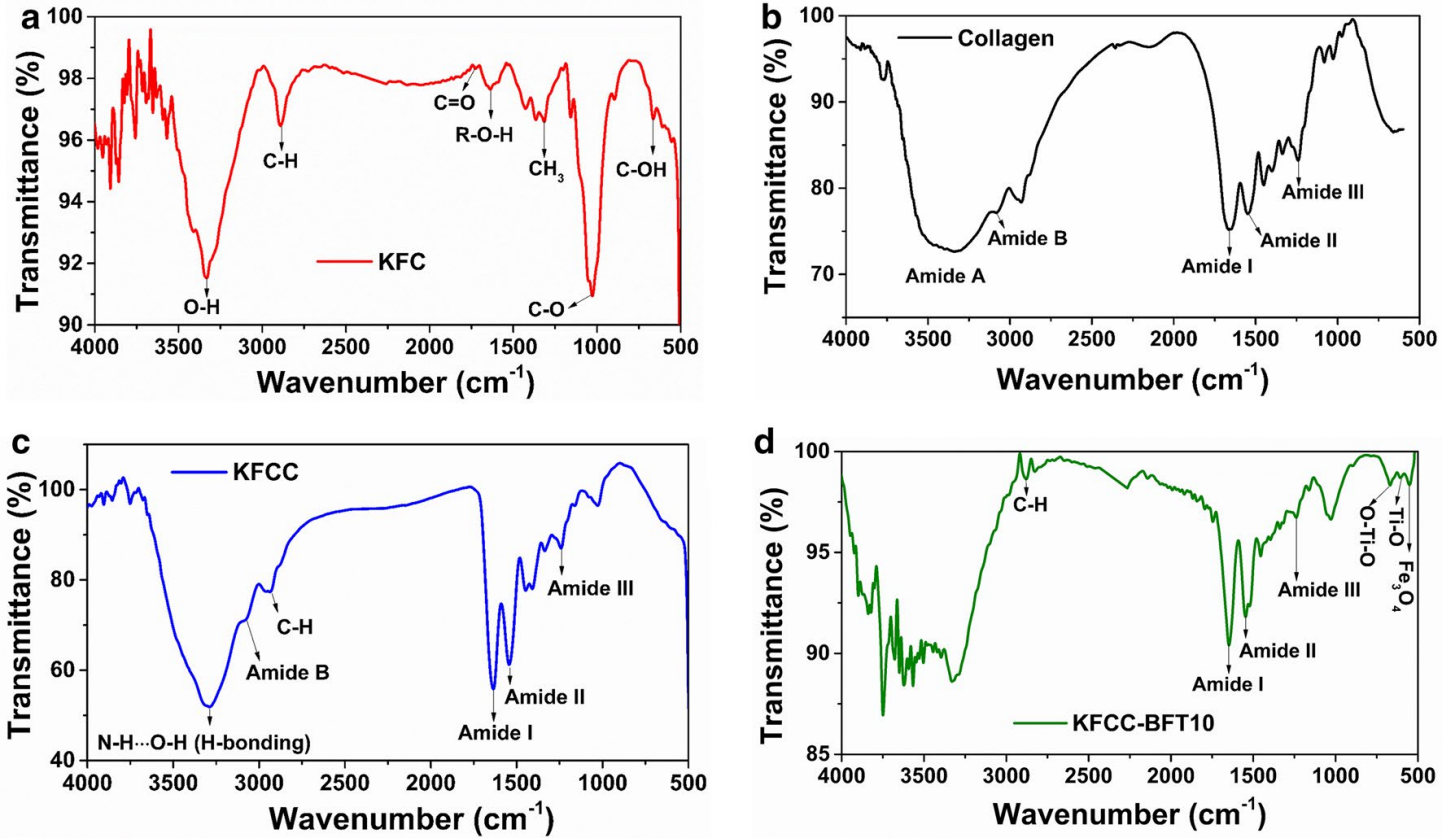
FT-IR spectra of (**a**) KFC, (**b**) Collagen, (**c**) KFCC, and (**d**) KFCC-BFT10.

FTIR spectrum of the extracted cellulose from kenaf fiber is shown in Fig. 3a. The band at 3336 cm^−1^ is attributed to the O–H stretching vibration and hydrogen bond of the hydroxyl groups. The band at 2889 cm^−1^ is the characteristic peak for alkanes exhibiting C–H stretching vibration of CH and CH_2_ asymmetric stretching in the cellulose backbone. The adsorbed water binding H–O–H band is observed at 1636 cm^−1^. The absorption peak at 1341 cm^−1^ corresponds to CH_3_ deformation. The intense band, centered at 1031 cm^−1^ is associated with the C–O stretching modes of the hydroxyl and ether groups in the cellulose. The band at 900 cm^−1^ can be attributed to the presence of β-glycosidic linkages and the small peak at 666 cm^−1^ is associated with the C–OH out-plane bending. It is interesting to note the disappearance of characteristics peaks at 1725 cm^−1^ and 1505 cm^−1^ corresponding to C=O stretching vibration of the acetyl groups in the hemicelluloses and the C=C stretching of the benzene ring of the lignin, respectively, indicating successful removal of these components during the cellulose extraction process.

The FT-IR spectrum of pristine collagen in Fig. 3b depicts the characteristics peaks including the amide I (C=O stretching) at 1664 cm^−1^, the amide II (N–H bending couple to C–N vibration) at 1546 cm^−1^, the amide III (C–N stretching and N–H bending) at 1244 cm^−1^, the amide A (C–N stretching and N–H bending vibration) at 3343 cm^−1^ and the amide B (C–H stretching) at 3081 cm^−1^^6^. The KFCC bio-sponge spectrum shows (Fig. 3c) characteristic peaks for both pristine collagen and cellulose with few changes in some peaks. The broad band at 3343 cm^−1^ corresponds to the N–H stretching vibration of amide A of collagen changed as a sharp band and present at the lower wavelength of 3322 cm^−1^. This result suggests that the N–H group of collagen may be involved in hydrogen bonds^43^. Interestingly, the characteristics peaks of pristine collagen corresponding to the amide I group shifted from 1664 to 1644 cm^−1^, from 1546 to 1536 cm^−1^ for the amide II group, and the amide III group from 1244 to 1237 cm^−1^, thus, indicating a blue shift of 20 cm^−1^, 10 cm^−1^ and 7 cm^−1^, respectively. These downshifts indicate a possible hydrogen bond interconnection between N–H groups of collagen and the hydroxyl OH group of cellulose^43^.

In the FTIR spectrum (Fig. 3d) of the magnetic hybrid bio-sponge KFCC-BFT10, we can observe key characteristic peaks of pure collagen and cellulose. Meanwhile, new peaks are observed at 549, 583 and 660 cm^−1^ corresponding to Fe–O stretching vibration of magnetite Fe_3_O_4_, Ti–O, and O–Ti–O bonds, respectively^5,12^. The presence of oxide bond peaks in the bio-sponge indicates the successful incorporation of the metal nanoparticles in the organic sponge.

**Figure 4:**
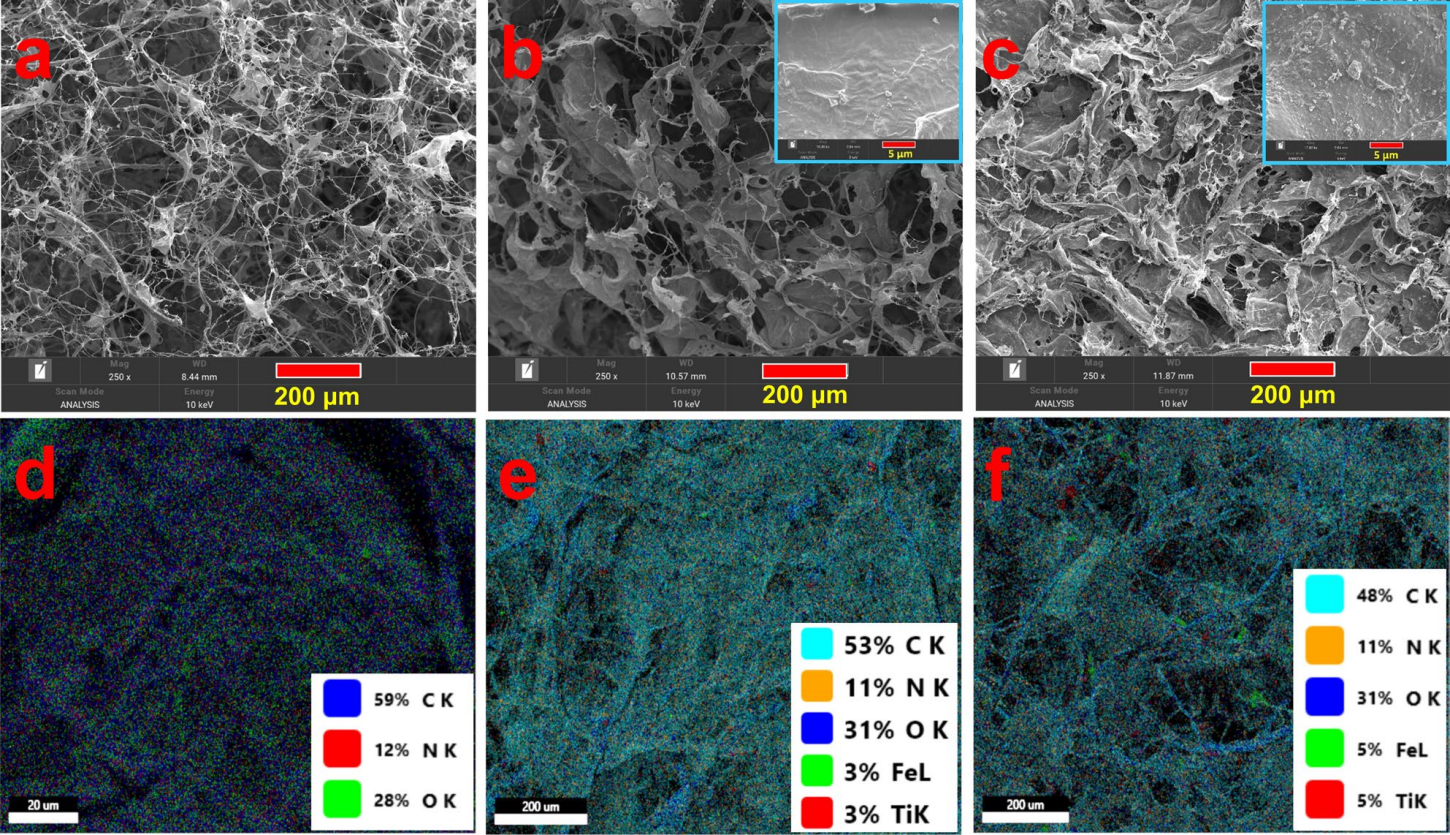
FESEM images of as-synthesized (**a**) KFCC, (**b**) KFCC-BFT5 and (**c**) KFCC-BFT10 bio-sponges and inserts show magnified KFC-BFT5 and KFC-BFT10 sponges at 5 μm scale. Elemental mapping micrographs of as-synthesized (**d**) KFCC, (**e**) KFCC-BFT5 and (**f**) KFCC-BFT10 bio-sponges.

The surface morphology of the bio-sponges (Fig. 4a–c) shows a multi-layered sheath-like spongious architecture and a highly linked porous network structure when probed through FESEM (Fig. 4a–c). It is clearly shown from the insert images (5 μm) that the hybrid bio-sponges (Fig. 4b and c) depict relatively dense and compact structures compared to the pristine KFCC bio-sponge (Fig. 4a). Further, it can be seen from Fig. 4c that the bimetallic Fe_3_O_4_@TiO_2_ NPs are tightly anchored to the surface and interior of the bio-sponge fibers possibly through hydrogen bond interaction with Fe–OH or Ti–OH^25^. In comparison to the KFCC bio-sponge (Fig. 4d), the EDX mapping images show that both organic (C, N, O) and inorganic (Ti, Fe) elements are present in the hybrid bio-sponges (Fig. 4e,f). To investigate the elemental composition, a selected region of the SEM image was taken and the EDX spectral analysis was carried out. EDX spectra results displayed in Figure S3a–c confirm the presence of Fe and Ti in the KFCC–BFT nanocomposites. The presence of C, N and O peaks indicates the main elements in the KFCC (Kenaf fiber and collagen) as shown in Figure S3a. As can be seen, the intensity of the Fe and Ti peaks is higher in the KFCC-BFT10 nanocomposite (Figure S3c) when compared to KFCC-BFT5 (Figure S3b) corresponding to the dosage of the BFT. There were no other peaks in the spectra that may be attributed to contaminants. These findings prove the high adhesion of Fe and Ti-based bimetallic nanoparticles to the KFCC surface.

**Figure 5:**
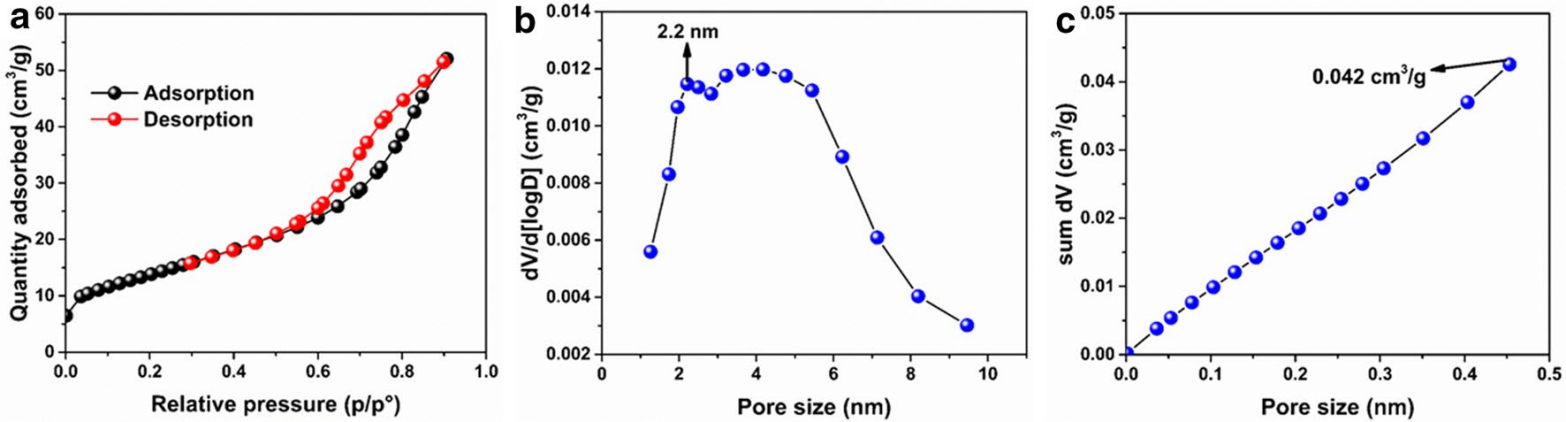
(**a**) BET plot (N_2_ adsorption–desorption isotherm) for KFCC-BFT10 bio-sponge, (**b**) BJH pore size distribution plot for KFCC-BFT10 bio-sponge. (**c**) BJH pore volume plot for KFCC-BFT10 bio-sponge.

The N_2_ adsorption–desorption technique was employed to investigate the pore size, pore diameter and pore volume of the KFCC-BFT10 bio-sponge. As shown in Fig. 5a, KFCC-BFT10 bio-sponge exhibited characteristic hysteresis loop in the adsorption–desorption isotherm. According to IUPAC classification, the N_2_ adsorption–desorption isotherm of the KFCC-BFT10 bio-sponge show type IV isotherm pattern with H3 hysteresis loop, which means that the KFCC-BFT10 bio-sponge belongs to typical mesoporous material. The KFCC-BFT10 bio-sponge possesses a specific surface area of 40.9 m^2^/g according to Brunauer–Emmett–Teller (BET). Pore size distribution of the KFCC-BFT10 bio-sponge (Fig. 5b) shows two maxima. A very narrow peak at 2.2 nm and a broad pore size distribution from 3 to 6 nm indicate fairly wide distribution of pores. The prepared KFCC-BFT10 bio-sponge shows pore volume of 0.042 cm^3^/g (Fig. 5c). The high surface area and pore volume obtained may provide adequate surface sites for increased photocatalytic activity and oil sorption.

**Figure 6:**
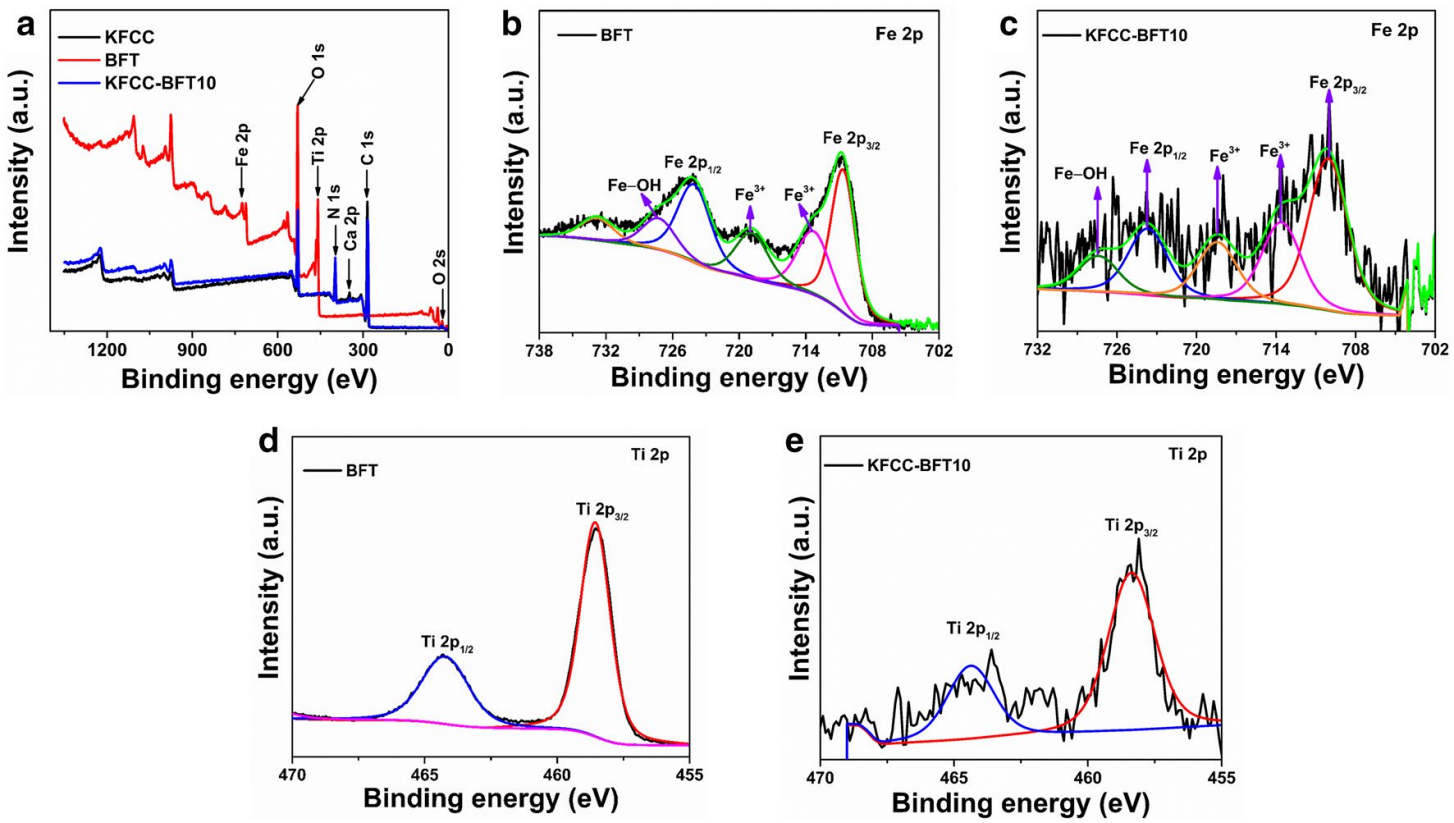
(**a**) XPS survey spectra of KFCC, BFT and KFCC-BFT10 bio-sponge. The Fe 2p core level deconvoluted XPS spectra of (**b**) BFT and (**c**) KFCC-BFT10. The Ti 2p core level deconvoluted XPS spectra of (**d**) BFT and (**e**) KFCC-BFT10.

**Figure 7:**
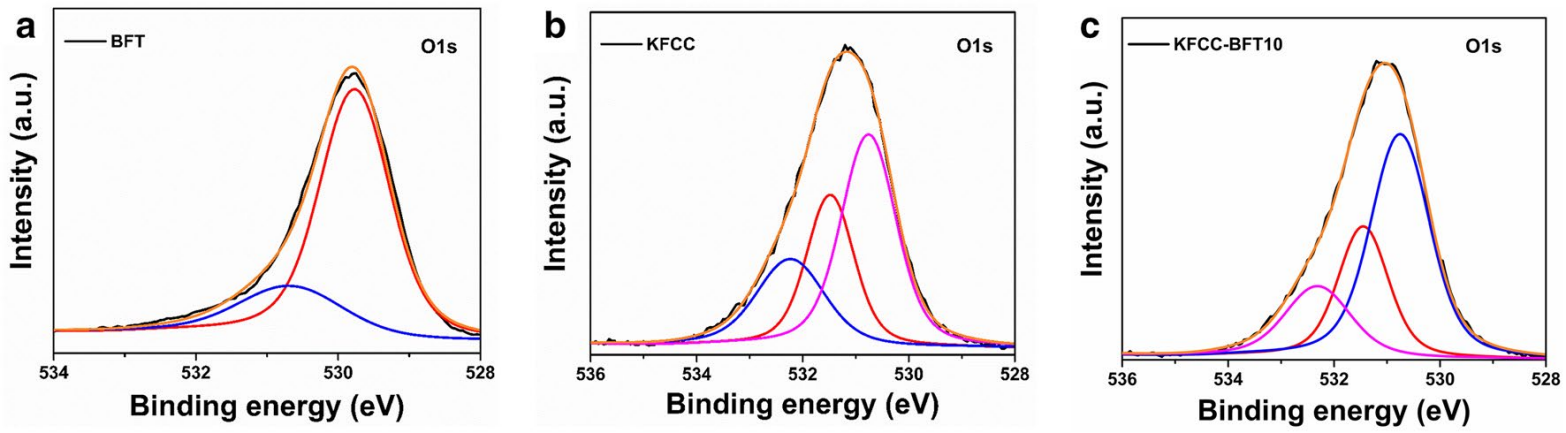
O 1s core level deconvoluted XPS spectra of (**a**) BFT, (**b**) KFCC and (**c**) KFCC-BFT10 bio-sponge.

The chemical composition and electronic structure of the developed photocatalyst were determined by the XPS spectra. The XPS survey spectra of KFCC, BFT and KFCC-BFT10 bio-sponge are illustrated in Fig. 6a and the core level spectra of Fe 2p and Ti 2p are shown in Fig. 6b–e. As can be seen in Fig. 6a, Fe 2p, Ti 2p, C 1s, N 1s, O 1s, and O 2s peaks are visible in the spectra of pristine BFT, KFCC and KFCC-BFT10 bio-sponge. These elemental compositions obtained for the nanoparticles, hybrid biopolymer matrix and nanocomposites through XPS analysis are concurrent with the FESEM − EDX data and further confirm that the samples are devoid of any impurities. The pristine BFT and KFCC-BFT10 bio-sponge showed two distinctive peaks of Fe^3+^ 2p_3/2_ and Fe^3+^ 2p_1/2_ in the Fe 2p spectra at 710.6 and 724.1 eV, respectively (Fig. 6b and c). It shows that the predominant form of Fe species in the nanocomposites is Fe^3+^ state. Additionally, the binding energies at 713.7 and 718.4 eV in the Fe 2p spectra of pure BFT could be attributed to Fe^3+^ in the spinel structure and the binding energy at 727.4 eV could be attributed to Fe^3+^ bound with a hydroxyl group. However, the binding energy in the Fe 2p spectra of the KFCC-BFT10 bio-sponge is slightly changed to 713.4, 719.0, and 727.5 eV, respectively. This meant that in the KFCC-BFT10 bio-sponge, the electrons could have more influence towards Fe^3+^. In the Ti 2p core level spectra of the pristine BFT10 and KFCC-BFT10 bio-sponge (Fig. 6d and e), two unique peaks at 458.4 and 464.3 eV are seen, which are attributed to Ti 2p_3/2_ and Ti 2p_1/2_, respectively, indicating that the titanium is mainly presented as Ti^4+^ in the anatase TiO_2_ form. This is in concurrence with our XRD data and also various literature values^44,45^. Thus, it is determined that Fe in Fe_3_O_4_@TiO_2_ has an oxidation state of + 3, whereas Ti is present as tetravalent and therefore both the Fe^3+^ and Ti^4+^ are in oxide form rather than in pure metallic form. The O 1s core level XPS spectra of pristine BFT, KFCC and KFCC-BFT10 bio-sponges are shown in Fig. 7. The binding energy range for the O 1s peak is 528–534 eV. Oxygen can exist in two different molecular states namely O^2−^ and O_2_^−^ with binding energy values of about 530 and 531.5 eV, respectively. For KFCC and KFCC-BFT10 bio-sponge, the O 1s peak appears at 530.7 and 531.5 eV, respectively. This component typically results from surface oxygen that is non-stoichiometric or OH from air. The surface O_2_ present in the oxides is responsible for the existence of a peak at 532.2 eV. In the case of BFT, two peaks are observed in the core-level spectra of O 1s. The low binding energy component observed at 529.7 eV is attributed to the O^2−^ forming oxide whereas the peak at 530.8 eV is assigned to OH^−^. As can be seen, XPS data show that iron is present in the chemical state of Fe^3+^ and oxygen is predominantly in the state of O^2−^, thereby verifying the formation of Fe_2_O_3_. In brief, the Fe element in the KFCC-BFT10 bio-sponge is more positively charged, which would help in the regeneration of Fe^3+^ and thus may stimulate the photocatalytic degradation reaction.

**Figure 8:**
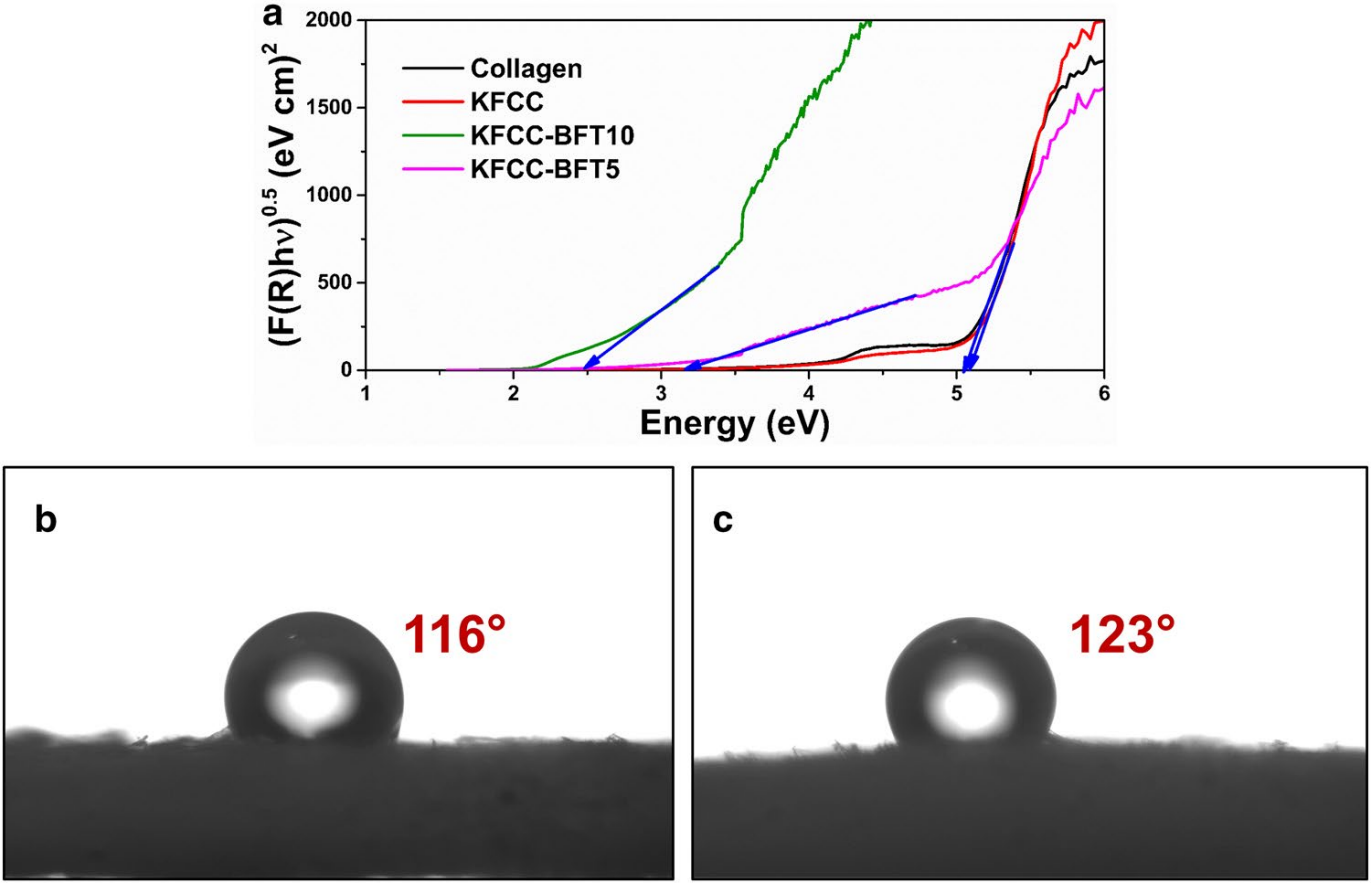
(**a**) UV–visible diffuse reflectance spectra of collagen, KFCC, KFCC-BFT5 and KFCC-BFT10 bio-sponges. Water contact angle of hybrid (**b**) KFCC-BFT5 and (**c**) KFCC-BFT10 bio-sponges.

The band gap energy of as-prepared bio-sponges was also investigated. From Fig. 8a, it is seen that the band gap energy of collagen and KFCC sponges are similar around ~ 5.1 eV and much higher than that of the hybrid KFCC-BFT10 bio-sponge, which is ~ 2.5 eV. It is seen that the band gap energy of bio-sponge without bimetallic nanoparticles is double the band gap energy of KFCC-BFT10. The possible reason for the decrease in band gap energy could be the effective interactions of Ti–O–Fe bonds in the KFCC matrix^5,17^. This reduced bandgap energy along with the red-shifted absorption in the visible region indicates that the hybrid KFCC-BFT10 bio-sponge can be excited under visible light or direct sunlight illumination to produce more electron–hole pairs for enhanced photocatalytic degradation activity of organic pollutants.

The water contact angles of the hybrid bio-sponges are shown in Fig. 8b and c and relatively high water contact angle values were achieved after the interaction with the bimetallic nanoparticles. As the percentage of bimetallic NP in the sponge increases, the water contact angle also increases. This finding suggests that the as-prepared bio-sponges are reasonably hydrophobic. On the other hand, during the oil contact angle measurement, the oil droplet penetrated quickly into the hybrid bio-sponges and the contact angle could not be recorded. The wettability tests prove that the hybrid bio-sponges exhibit selective wettability towards water and oil. These hybrid bio-sponges can easily adsorb oil and repel water. The water contact angle for the pristine collagen–cellulose (KFCC) sponge could not be recorded because the water droplet was immediately absorbed by the sponge due to the hydrophilic property of cellulose and collagen.

### Photocatalytic activity

**Figure 9:**
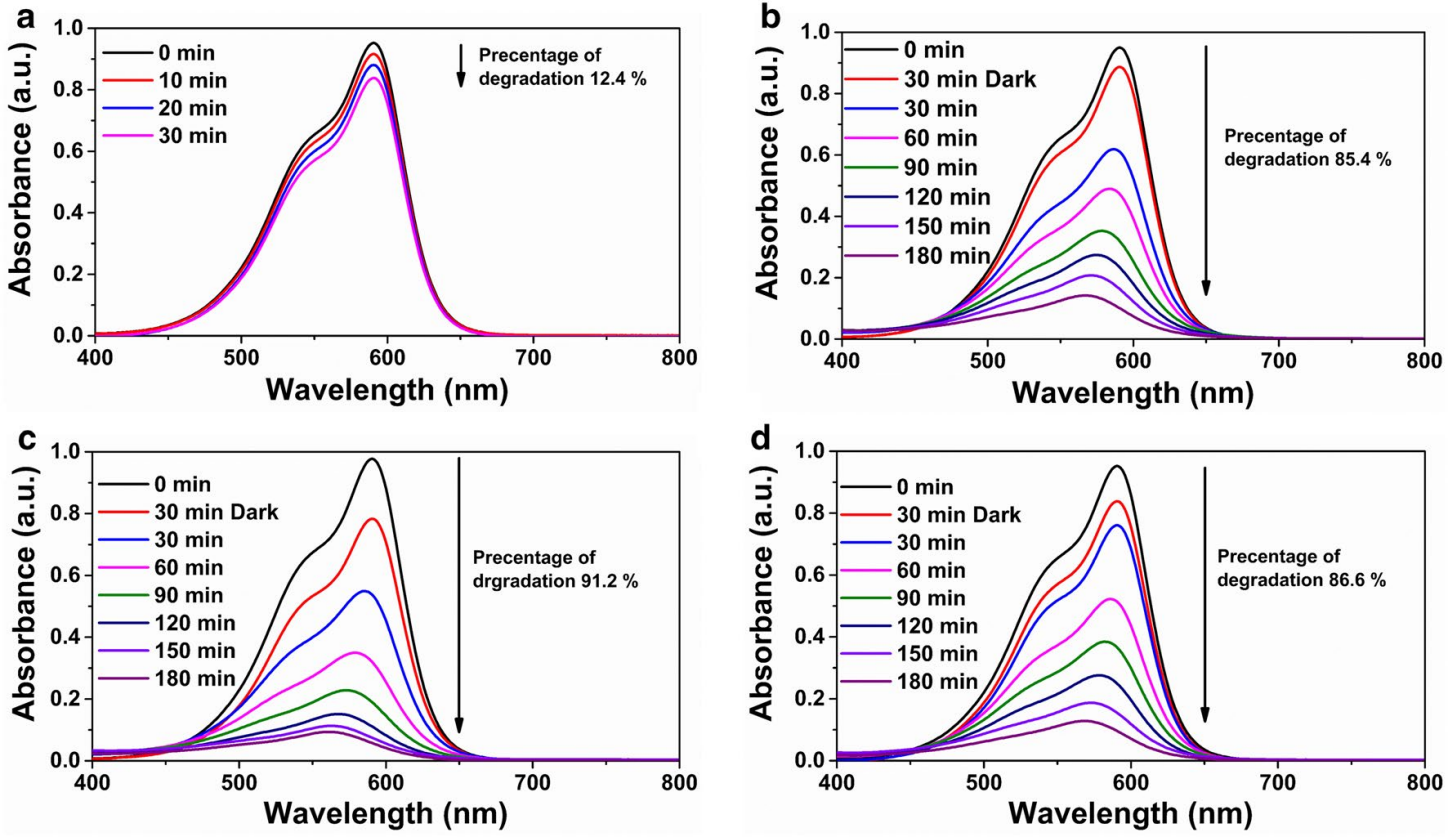
Photocatalytic degradation of crystal violet with (**a**) KFCC-BFT10 in dark condition, (**b**) KFCC-BFT5 and (**c**) KFCC-BFT10 in visible light irradiation and (**d**) KFCC-BFT10 under sunlight irradiation.

As-prepared bio-sponges were used to study the degradation of aqueous crystal violet (CV) dye under visible light and sunlight in the presence of H_2_O_2_. Hydrogen peroxide plays a vital role in the degradation of CV by the generation of two strong hydroxyl radicals (HO^·^)^46–48^. The mechanism for the degradation of CV in presence of magnetic bimetallic Fe_3_O_4_@TiO_2_ NPs has been described by Vinosel et al.^5^ As seen in Fig. 9a, the adsorption capacity of the bio-sponge is primarily responsible for the removal of roughly 12.4% of the CV in 30 min under dark conditions, which was realised until adsorption equilibrium is established. It is observed that the sharpness and the intensity of the maximum absorption peak of CV at 590 nm decreased with extended visible illumination time followed by slight blue-shifting for the samples treated with KFCC-BFT5 and KFCC-BFT10 bio-sponges (Fig. 9b and c).

**Figure 10:**
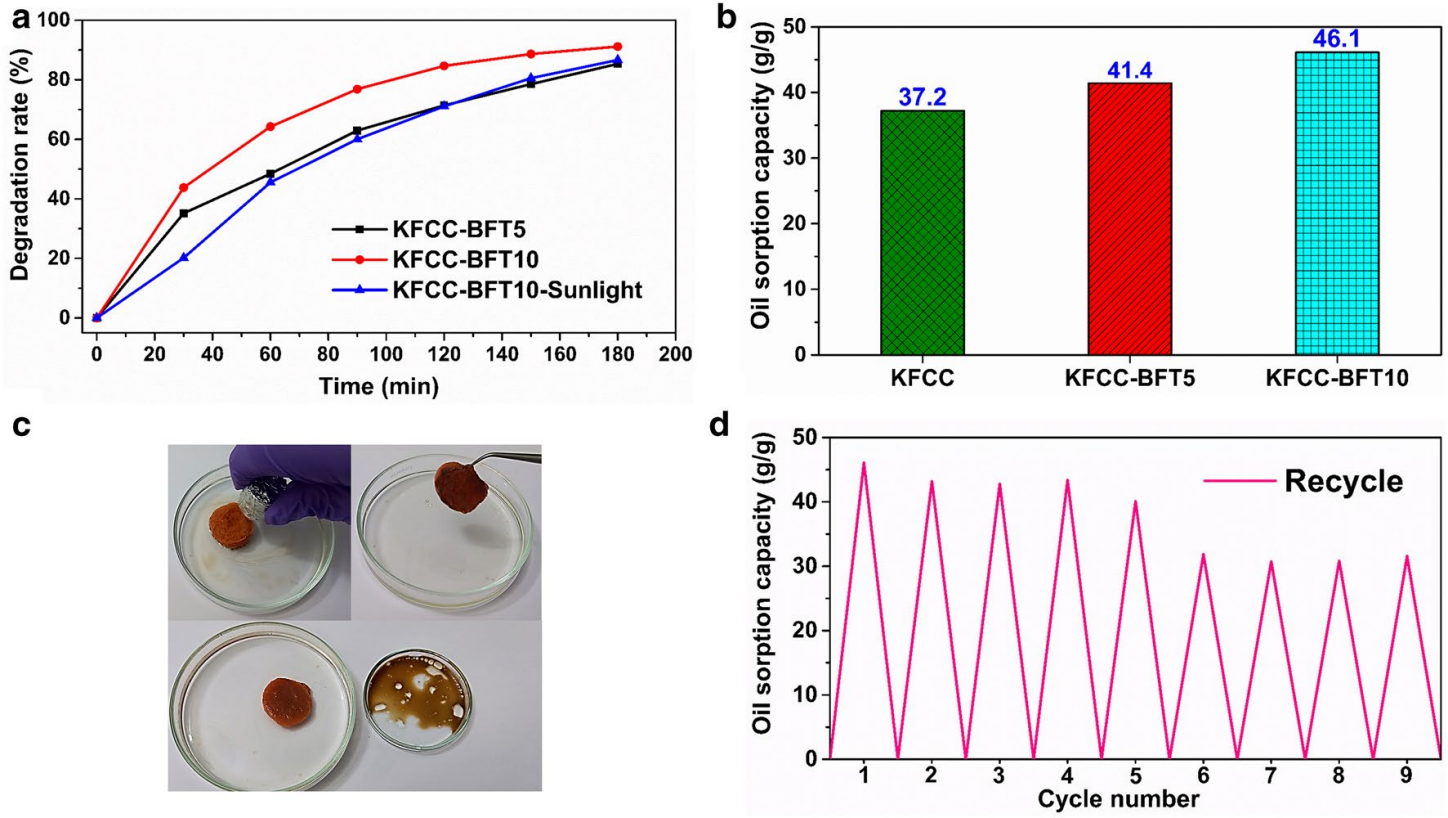
(**a**) Degradation rate of crystal violet dye as a function of time. (**b**) Oil sorption capacity of bio-sponge and (**c**) Digital photographs demonstrating the removal of oil using the hybrid bio-sponge from oil–water mixture. (**d**) Recyclability of the hybrid bio-sponge for oil removal.

The photocatalytic degradation efficiency of CV dye under visible light irradiation is 85.4 and 91.2% for KFCC-BFT5 and KFCC-BFT10 bio-sponges, respectively. After 1 h of irradiation (Fig. 10a), about 50% of CV dye has been degraded and more than 60% dye in the case of KFCC-BFT10 bio-sponge. The hybrid KFCC-BFT10 bio-sponge photocatalyst shows greater degradation efficiency of 91.2% compared to 86% using 500 mg of pure Fe_3_O_4_@TiO_2_ powder photocatalyst in 500 mL water as reported by Vinosel et al.^5^ As can be seen, KFCC-BFT10 bio-sponge exhibited higher degradation capability owing to the higher BFT content and hence further subjected to photocatalytic degradation under sunlight exposure. The results demonstrate 86.6% degradation of CV dye for KFCC-BFT10 bio-sponge under sunlight (Fig. 9d). As shown in Table 1, the photocatalytic degradation of the KFCC-BFT10 bio-sponge photocatalyst synthesised in this study is comparable to other similarly reported photocatalysts with the added advantage of easy biodegradability of the photocatalyst support. The better photocatalytic degradation obtained with KFCC-BFT10 bio-sponge can be explained by the 3D macroporous structure and the floatability behavior (low density ~ 9 mg.cm^−3^), which provide the photocatalyst the ability to ease photocatalytic oxygenation activity in the air–water interface with high induced photon and increased interaction between the hybrid magnetic NPs catalyst and CV dye due to the presence of BFT on the surface as well as the inner layers of the bio-sponge support.

**Table 1:** Comparison of different similarly reported photocatalysts and the KFCC-BFT10 bio-sponge reported in this study on the degradation performance towards dyes.

Target dye	Light source	Photocatalyst	Photocatalyst concentration (mg)	Degradation time (min)	Degradation efficiency (%)	References
Reactive Black	UV-C lamp	TiO_2_ on the Fe/Activated carbon	60	30	95	^49^
Methylene blue & Rhodamine B	simulated solar light (xenon lamp)	Ag − Fe_3_O_4_@TiO_2_	20	90	~ 100	^50^
Malachite green & Methylene blue	Visible light (Xenon lamp)	rGO-Fe_3_O_4_/TiO_2_	15	55	99	^51^
Methylene blue	UVA light	Fe_3_O_4_/TiO_2_-graphene quantum dots	400	90	86	^52^
Rhodamine B	Solar simulator	Fe_3_O_4_/TiO_2_	30	35	99.6	^53^
Crystal violet	Visible light (200 W Hg (Xe))	Cellulose–Collagen–Fe_3_O_4_@TiO_2_	30	180	~ 100	This study

### Oil removal efficacy

To evaluate the sorbent performance characteristic of bio-sponge as a bio-based, easily biodegradable and eco-friendly material for oil spill removal (Fig. 10b–d), the oil adsorption capacity was investigated. From Fig. 10b, it is clearly seen that both hybrid bio-sponges exhibit a higher adsorption capacity of 41.4 and 46.1 g/g for KFCC-BFT5 and KFCC-BFT10 bio-sponges, respectively, compared to KFCC bio-sponge (37.2 g/g). This high adsorption capacity of the hybrid sponge can be corroborated by its 3D macroporous network structure and hydrophobicity. An added advantage of the developed magnetic hybrid bio-sponge is the ability to track the motion of the sponge on the surface of oil–water emulsion under a magnetic field (Fig. 10c). It is well established that the oil sorption capacity of a sorbent is mainly governed by some physical sorption properties such as the capillary effect due to the porous structure, van der Waals forces and the hydrophobic interactions between the sorbent and oils^54^, and in the present case the tracking under magnetic field is considered as an additional factor that controls the adsorption capacity of sorbent.

The recyclability and reusability of sorbent after oil removal is an important aspect to be considered during the evaluation of performance characteristics of an adsorbent such as adsorption capacity after repeating cycles of adsorbing-removing and waste management of the sorbent after being used in the environment. The as-prepared sponges are green and easily biodegradable and thus waste management after use can be done naturally without causing any harm to human beings and the environment. The recyclability and reusability data reveal that the hybrid bio-sponge exhibits high sorption capacity even after 5 cycles, showing only a small decrease from 46.1 g/g to around 40 g/g, after which the oil sorption efficiency decreases up to around 30 g/g and remains relatively constant up to 9 cycles (Fig. 10d). During recycling, it has been noticed that the hybrid bio-sponge still contains trapped oil rendering it sticky, highly packed, and dense material after centrifugation. After each cycle, the sponge was immersed in ethanol solution 2 times for 1 h, freeze-dried and lyophilized for 24 h. Figure 10c shows that the adsorbed oil may be easily removed by squeezing out and only a small amount of oil is left in the KFCC-BFT10 nanocomposite. The KFCC-BFT10 nanocomposite could be easily separated from the oil/water mixture by forceps and used for the next run thereby demonstrating its potential for oil removal applications with recyclability.

## Conclusion

In summary, an easily biodegradable collagen–cellulose-based magnetic bio-sponge loaded with bimetallic Fe_3_O_4_@TiO_2_ NPs was fabricated with high potency for dye and oil removal. The structural morphology analysis by HRTEM revealed no core–shell structure but the deposition of TiO_2_ nanoparticles onto the surface of magnetite Fe_3_O_4_ nanoparticles. From room temperature magnetization measurement, it is found that the saturation magnetization value of bio-sponge was decreased compared to the value of pure BFT NPs and hysteresis loops with an S-like curve indicating ferromagnetic behavior of the bimetallic nanoparticles and bio-sponge. The morphology of the magnetic hybrid bio-sponge showed a 3D macroporous interconnection network with the BFT NPs bound on the surface and interior of the collagen–cellulose fibrous network as visualized through HRSEM. The wettability tests proved that the incorporation of the bimetallic NPs enhanced the hydrophobicity and roughness of the hybrid bio-sponges. The as-prepared bio-sponge exhibited high photodegradation activity by the removal of crystal violet dye up to 91.2 and 86.6% under visible light and direct sunlight irradiation, respectively. It is also demonstrated that a high oil sorption capacity of 46.1 g/g was achieved and the bio-sorbent can be recycled and reused up to 9 cycles. Based on the results, the as-prepared magnetic hybrid bio-sponge has a dual potential ability in wastewater remediation for dye removal and oil spill management thereby suggesting effective visible-light-driven photocatalytic application at an industrial scale.

### Supplementary Information

Supplementary Information.

## Data Availability

The datasets generated during and/or analysed during the current study are available from the corresponding author on reasonable request.
