# Supplementary material for: Hybrid collagen–cellulose–Fe3O4@TiO2 magnetic bio-sponges derived from animal skin waste and Kenaf fibers for wastewater remediation
Source: Sci Rep. 2023 Aug 17;13:13365. doi: 10.1038/s41598-023-40520-y (PMC10435533; doi:10.1038/s41598-023-40520-y)
Supplement: Supplementary file 1 — Supplementary Information. [file 41598_2023_40520_MOESM1_ESM.docx]

**Supporting Information**

**Hybrid collagen-cellulose-Fe_3_O_4_@TiO_2_ magnetic bio-sponges derived from animal skin waste and Kenaf fibers for wastewater remediation**

**E. F. Assanvo^a,b^, S. Nagaraj^a,c^, D. Boa^b^** **and P. Thanikaivelan^a,c^***

*^a^Advanced Materials Laboratory, CSIR-Central Leather Research Institute (CSIR-CLRI), Sardar Patel Road, Adyar, Chennai 600 020, India*

*^b^Laboratoire de Thermodynamique et de Physico-Chimie du Milieu, UFR SFA, Université Nangui Abrogoua, 02 BP 801 Abidjan 02, Côte d’Ivoire*

*^c^University of Madras, Chepauk, Chennai 600005, India*

^*^Author to whom correspondence should be made.

Tel: +91 44 24437142

Email: thanik8@yahoo.com; thanik@clri.res.in

†Electronic supplementary information (ESI) available. See DOI: xxxxx


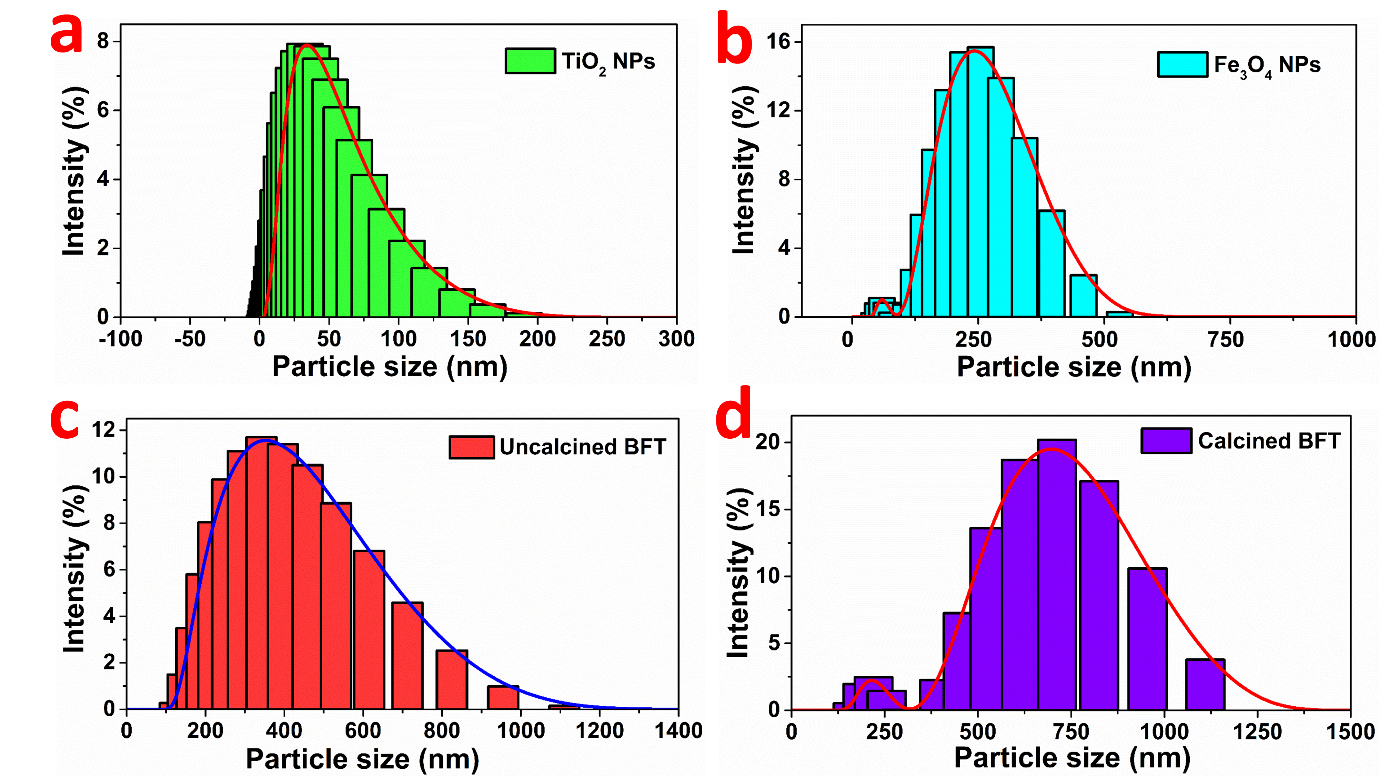


**Figure S1.** Particle distribution of (a) TiO_2_ NPs, (b) Fe_3_O_4_ NPs (c) uncalcined magnetic BFT NPs and (d) magnetic BFT NPs after calcination.


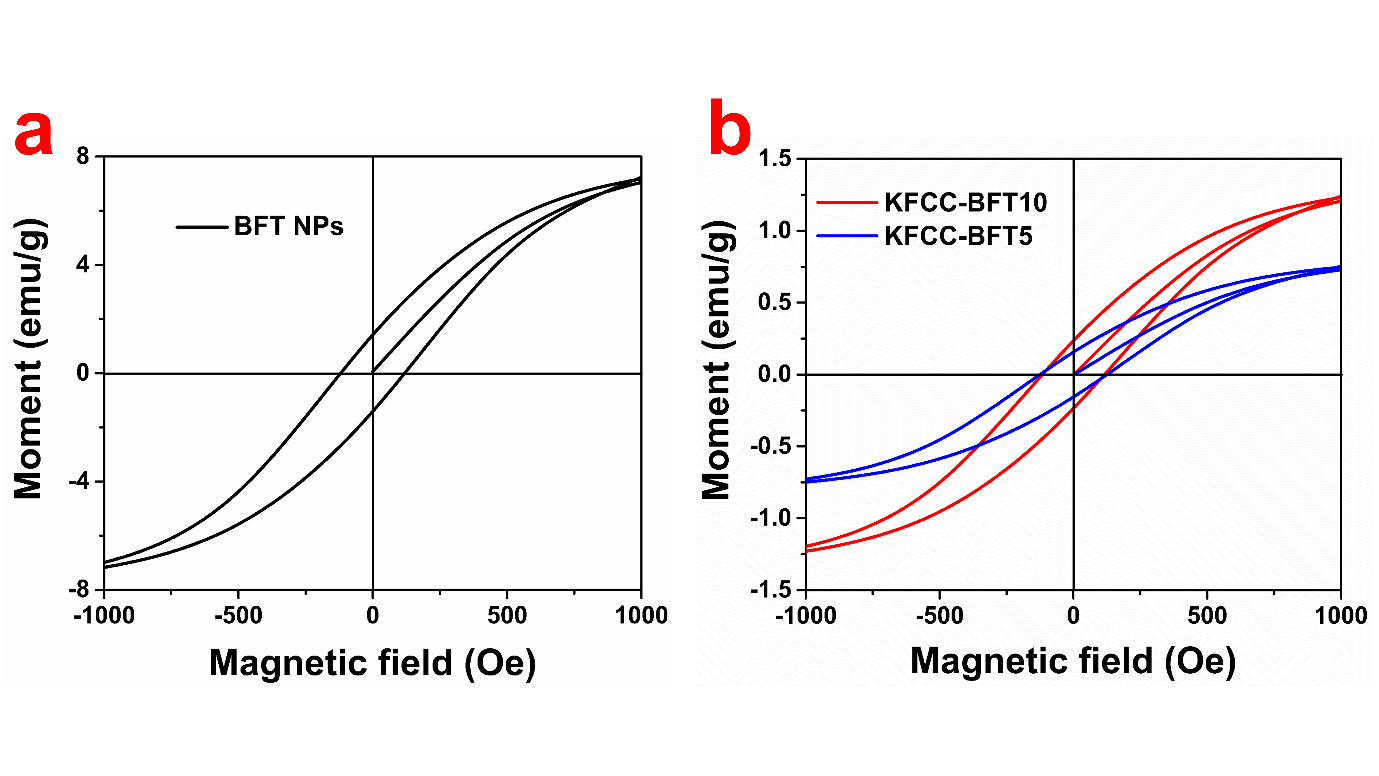


**Figure S2.** Room-temperature hysteresis loops of as-synthesized (a) BFT NPs and (b) KFCC-BFT5 and KFCC-BFT10 bio-sponges.

**
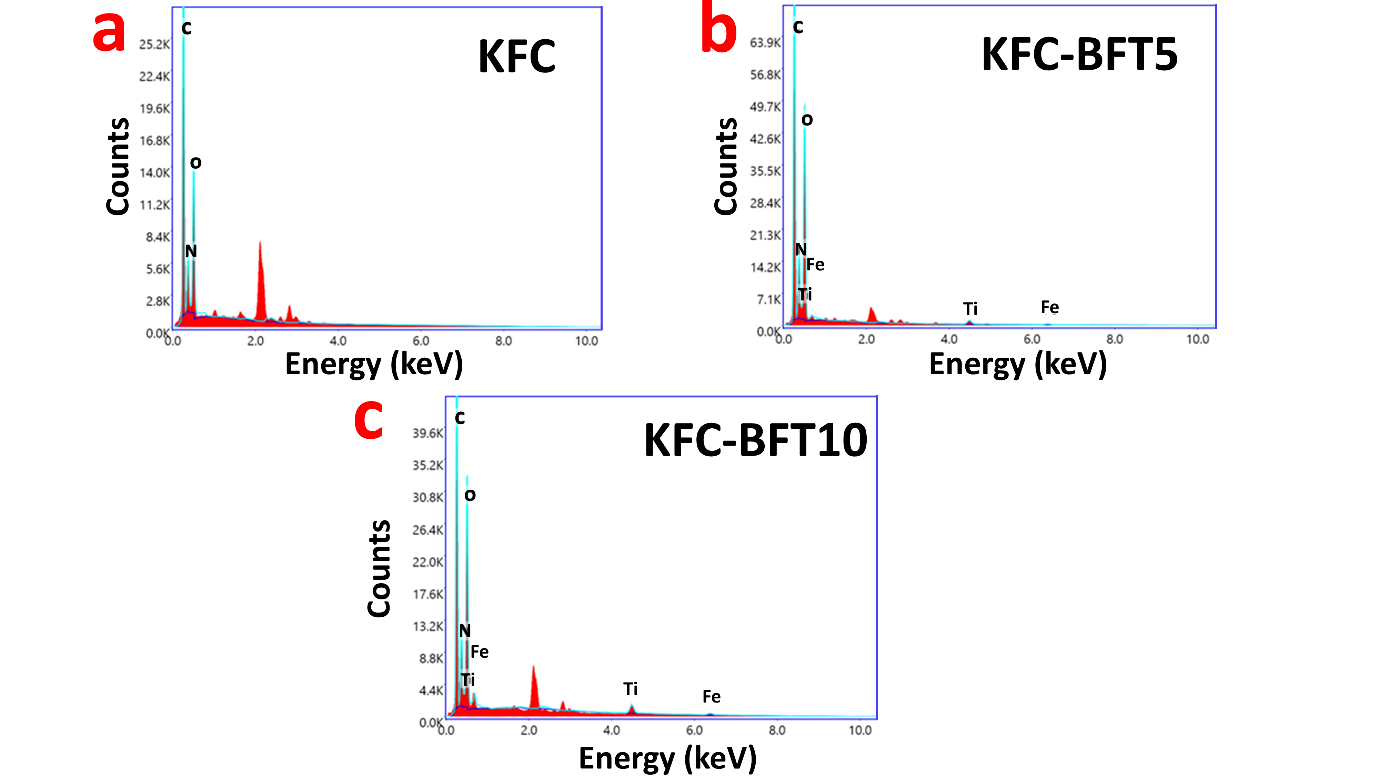
**

**Figure S3.** EDX spectra showing the elemental composition (iron, titanium, carbon, oxygen,

nitrogen) of (a) KFC, (b) KFC-BFT5 and (c) KFC-BFT10.
